# Dynamic Thermal Relaxation in Metallic Films

**DOI:** 10.3390/e28080908

**Published:** 2026-08-13

**Authors:** Libin Wang, Dmitry Golubev, Yuri M. Galperin, Jukka P. Pekola

**Affiliations:** 1 QTF Centre of Excellence, Department of Applied Physics, Aalto University, FI-00076 Espoo, Finland; 2 HQS Quantum Simulations GmbH, Rintheimer Str. 23, 76131 Karlsruhe, Germany; 3 Department of Physics, University of Oslo, Blindern, P.O. Box 1048, 0316 Oslo, Norway

**Keywords:** Josephson junction thermometer, thermal relaxation, heat capacity

## Abstract

The performance of low-temperature detectors utilizing thermal effects is determined by their energy relaxation properties. Usually, heat transport experiments in mesoscopic structures are carried out in the steady state, where temperature gradients do not change in time. Here, we present an experimental study of dynamic thermal relaxation in a mesoscopic system—thin metallic film. We find that thermal relaxation of hot electrons in copper and silver films is characterized by several time constants, and that the annealing of the films changes them. In most cases, two time constants are observed, and we can model the system by introducing an additional thermal reservoir coupled to the film electrons. We determine the specific heat of this reservoir and its coupling to the electrons. We suspect that multiscale thermal relaxation arises from the complicated morphology of the films, in which the electron–phonon coupling strength in grains with different orientations varies.

## 1. Introduction

Investigation of thermal transport in mesoscopic devices is essential for the study of fundamental physics and for the development of low-temperature detectors [1]. Examples include transition edge sensors [2,3,4,5,6], hot-electron detectors [7,8,9,10,11,12] and other types of nano-calorimeters in the application of photon and particle detection [13,14,15,16], mass spectrometry [17,18] and in the field of quantum technologies [19,20,21,22]. In the steady state, thermal transport down to a single heat-conducting channel has been studied in recent decades [23,24,25,26,27]. In contrast, experiments carried out in the dynamic regime are rarely reported, mainly due to the lack of fast and sensitive thermometers to measure system temperature. Recently, several types of fast thermometers have been developed, which have made it possible to investigate the dynamic thermal relaxation in mesoscopic systems [28,29,30,31,32]. Such experiments are particularly important for low-temperature detectors utilizing thermal effects. Indeed, thermal relaxation determines the performance of the detector and its recovery time after a detection event. In the absence of detailed time-resolved measurements, the relaxation in the detector is usually assumed to be exponential, and its time constant is estimated from the parameters measured in the steady state [29,33]. Here we demonstrate the deficiency of this approach for a metallic film absorber, which may exhibit several relaxation times and may equilibrate very slowly.

**Figure 1:**
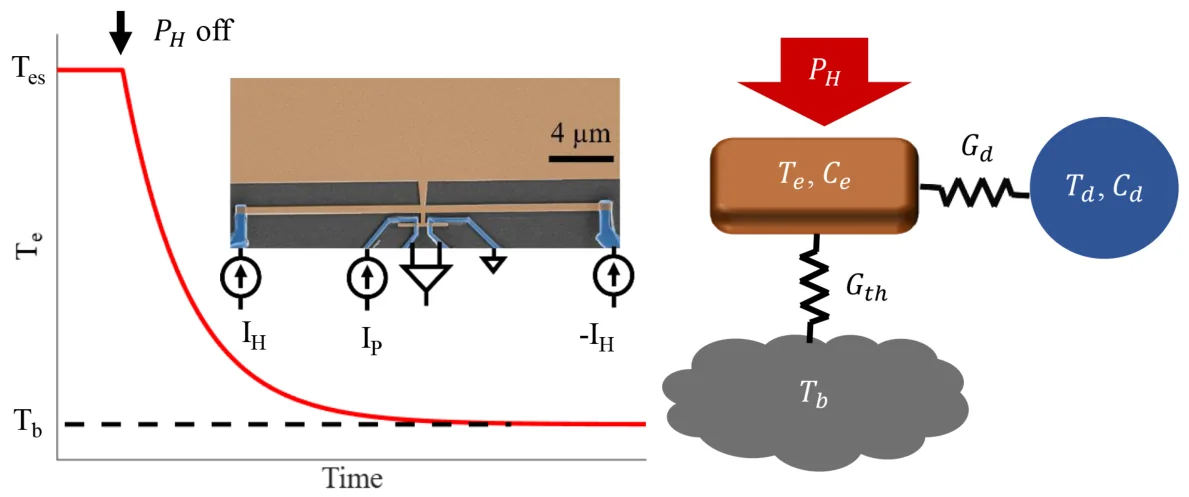
(**Left panel**): Time dependence of the electron temperature $T_{e}$ in response to the external heating power $P_{H}$. After $P_{H}$ is switched off, the electron temperature relaxes to the bath temperature $T_{b}$. Inset: False-color SEM image of a typical sample with the measurement circuits (Cu is shown in brown, Al in blue). (**Right panel**): Thermal model of a heated Cu film on a dielectric substrate. Film electrons are thermally coupled to the bath ($T_{b}$) and to an additional thermal reservoir ($T_{d},C_{d}$) by thermal conductances $G_{th}$ and $G_{d}$.

In this article, we mostly focus on the thermal relaxation process in a copper (Cu) film and study the time dependence of the electron temperature after the applied heating is turned off, as shown in Figure 1. Theory predicts that for a normal metal at low temperatures, thermal relaxation is governed by electron–phonon (e-ph) coupling [34], and then the relaxation time $\tau_{e-ph}=\gamma /5\Sigma T^{3}$. Here γ is the Sommerfeld constant, which determines the heat capacity of electrons; $C_{e}=\gamma VT$, with *V* being the volume of the film; and Σ is the characteristic electron–phonon coupling constant defining the heat flux $P_{e-ph}$ between electron and phonon sub-systems with the temperatures $T_{e}$ and $T_{ph}$; $P_{e-ph}=\Sigma V\left(T_{e}^{5}-T_{ph}^{5}\right)$. For Cu, these two parameters take the values γ = 98 ${\mathrm{JK}}^{-2}$$m^{-3}$ [35] and Σ = 2 ${\mathrm{nWK}}^{-5}$μ$m^{-3}$ [34,36], which results in a relaxation time of $\tau_{e-ph}∼10\;$ μs at 100 mK. In our experiment, we show, contrary to theoretical prediction, that the relaxation of electron temperature in Cu films is characterized by several time constants. We have observed the same effects in silver (Ag) films (See Supplementary Material). Our results suggest that this phenomenon is caused by the combined effect of strong anisotropy of electron–phonon scattering rate in bulk Cu and Ag [37,38,39,40,41] and of the complex texture of thin films, which contain grains with different orientations relative to the substrate [42,43].

## 2. Materials and Methods

One of our devices is shown as a false-color SEM image in the inset of Figure 1. Cu films of varying thicknesses are evaporated on ${\mathrm{SiO}}_{2}/\mathrm{Si}$ substrate with the electron-beam evaporation technique. Superconducting aluminum (Al) is used for the galvanic connection to the Cu films. The large pad on top (partly shown) constitutes the main volume of the film to be studied. The long horizontal Cu wire in the middle is a heater; it is biased with currents of opposite polarities $\pm I_{H}$. The short wire at the bottom, which contacts two aluminum leads at both ends, behaves as a proximity JJ. It is used as a fast thermometer to monitor electron temperature in the Cu film. In the explored temperature range, its resolution was around 0.1 mK for a probing pulse of 2 μs duration (See Supplementary Material). The sample is cooled down with a home-made wet dilution refrigerator, and it is shielded against the environmental radiation with a sample holder shield at low temperature. Details of the measurement technique have been reported previously [30]. Thermal equilibration times between the heater, the thermometer, and the pad can be estimated as $\tau_{eq}^{ij}=C_{i}/G_{ij}$, where $C_{i}$ is the heat capacity of the corresponding film and $G_{ij}$ is the heat conductance of the bridge connecting the two components. We find $\tau_{eq}^{ij}<10$ ns at 0.1 K for all measured devices, which is much shorter than thermal relaxation times (See Supplementary Material).

## 3. Results

**Figure 2:**
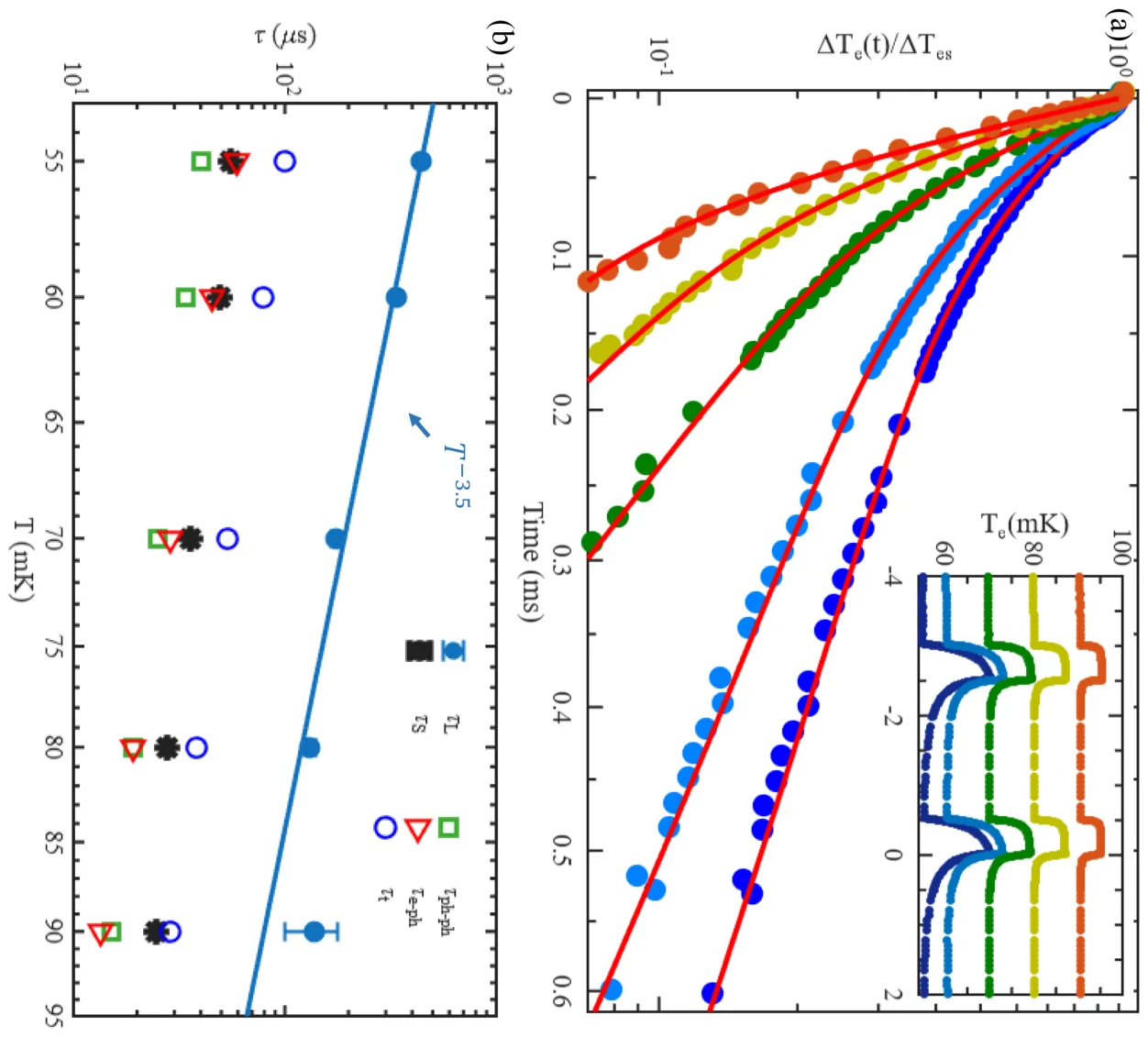
Thermal relaxation in a 300 nm thick Cu film. (**a**) Time dependence of the normalized electron temperature $\Delta T_{e}\left(t\right)/\Delta T_{es}$. Bath temperatures are $T_{b}$ = 55, 60, 70, 80, 90 mK from right to left. Red lines are fits with Equation (1). Inset: Electron temperature versus time. The values of $T_{b}$ are the same as in the main plot. (**b**) Temperature dependence of the two time constants $\tau_{L}$ and $\tau_{S}$. Error bars are smaller than the marker size for most points. $\tau_{e-ph}$, $\tau_{ph}$ and $\tau_{t}$ are the theory-predicted time constants given by Equation (4). The solid blue line shows a simple power-law fit of the dependence $\tau_{L}\left(T\right)$.

In the inset of Figure 2a, we show the time dependence of the electron temperature for the two heating current pulses with inverse polarities. Each current pulse has a width of 500 μs, and they were applied during the time intervals −3.5 to −3 ms and −0.5 to 0 ms. The thickness of the Cu film was 300 nm. Before the heating pulse was applied, i.e., for −4<t<−3.5 ms, the electrons were in thermal equilibrium, with the bath temperature $T_{b}$. After the heating was turned on, the electron temperature began to rise and finally reached the steady-state value $T_{es}$. Identical responses to the two heating pulses confirm the accuracy of our measurement technique. The steady-state temperature rise $\Delta T_{es}=T_{es}-T_{b}$ is consistent with the previous DC measurements [44]. It decreases at high $T_{b}$ due to the increase in the thermal conductance between the electrons in the film and the environment.

In order to investigate the dynamic thermal relaxation, we have recorded the time dependence of the electron temperature after the heating current is turned off. Figure 2a shows the normalized $\Delta T_{e}\left(t\right)$ as a function of the time *t*. We have found, contrary to our expectation, that the dependence $\Delta T_{e}\left(t\right)$ could not be fitted with an exponentially decaying function. However, we could very well fit the data with two exponents:

(1) $$\Delta T_{e}\left(t\right)/\Delta T_{es}=ae^{-\frac{t}{\tau_{L}}}+\left(1-a\right)e^{-\frac{t}{\tau_{S}}},$$

where *a* is a constant pre-factor and $\tau_{S}$ and $\tau_{L}$ are, respectively, the short and the long relaxation times. The nonlinear regression method has been used to fit the data with *a*, $\tau_{S}$, and $\tau_{L}$ as free parameters. Ordinary Least Squares (OLS) regression of the fits varied between 99.97–99.99%. The red lines in Figure 2a show the corresponding fits. The times $\tau_{S}$ and $\tau_{L}$ for different temperatures are shown in Figure 2b. At low temperatures, the time constant $\tau_{L}$ is about one order of magnitude longer than $\tau_{S}$, and at high temperatures, $\tau_{L}$ and $\tau_{S}$ differ even more. As a result, at high temperatures $\tau_{S}$ cannot be resolved, and the relaxation process takes a single exponential form with a time constant $\tau_{L}$. This explains previous observations of very long relaxation times in Cu and AuPd films [9,28]. Due to the high sensitivity of our JJ thermometer at low temperatures, we can now clearly distinguish the two time constants. Performing simple power-law fits, we find that the relaxation times scale with temperature as $\tau_{L}\propto T^{-3.5}$ and $\tau_{S}\propto T^{-3}$. We have also verified that the effect of the heating pulse amplitude on thermal relaxation is negligible for temperature increments within the range 6 mK $<\Delta T_{es}<$ 18 mK (See Supplementary Material).

**Figure 3:**
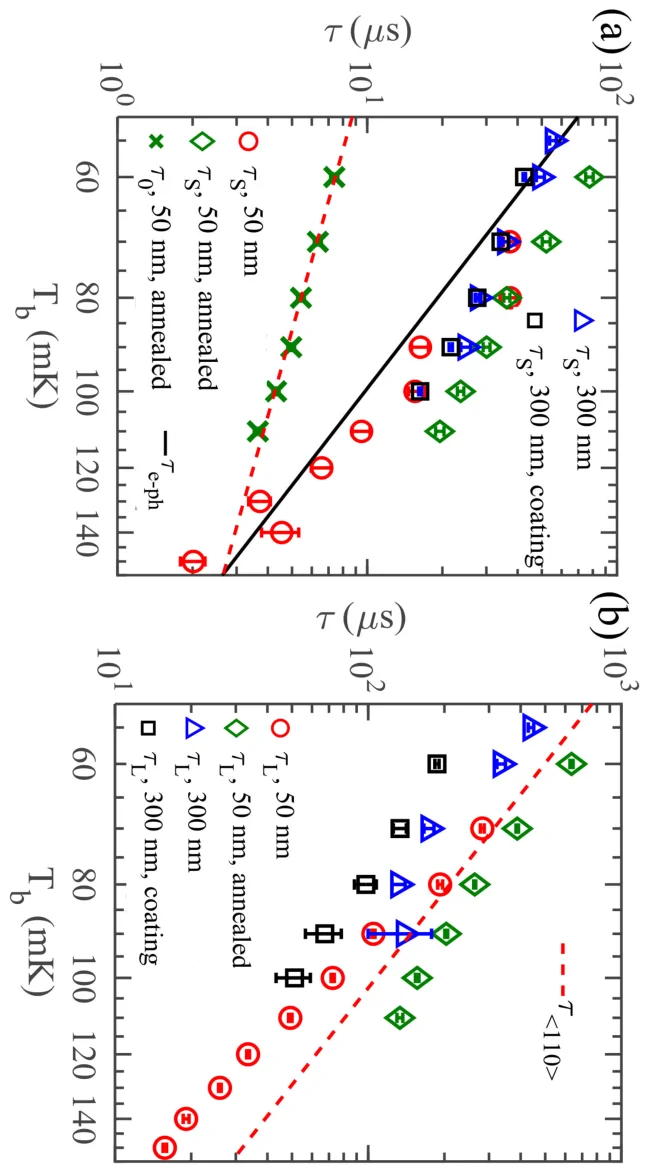
Temperature dependence of thermal relaxation times for all samples. (**a**) Short time constant $\tau_{S}$ (for the annealed 50 nm film, we show both $\tau_{S}$ and the shortest relaxation time $\tau_{0}$). Black solid line shows the calculated electron–phonon time $\tau_{e-ph}$ (4); red dashed line is the linear fit of $\tau_{0}$ of the annealed 50 nm film. (**b**) Longer relaxation time $\tau_{L}$ versus temperature. Red dashed line shows the electron–phonon relaxation time of electrons moving in <110> direction measured in bulk Cu, $\tau_{\langle 110\rangle }=10^{-7}\times {\left(T/1K\right)}^{-3}$ s [37].

We have repeated the measurements with several films to test the dependence of the relaxation times on the film thickness, which has been observed previously [45]. Such dependence would suggest that multiple relaxation times may be caused by impurities or defects on the surface of the film. To verify this, we have fabricated a sample with a film thickness of 50 nm, in which the surface-to-volume ratio is increased by a factor of 6, and a sample with a 300 nm Cu film coated with a 5 nm thin layer of Al right after evaporation. The volumes of these two films were the same as the volume of the original 300 nm film, which was 120 μ$m^{3}$. We have found that both films show two time constants, which are quite close to the ones of the 300 nm film without surface coating, as shown in Figure 3a. Finally, we have measured an annealed 50 nm thin Cu film in order to test the effect of the grain size and grain interface on thermal relaxation. SEM pictures of the sample show significant growth in the grain size after annealing (See Supplementary Material). Thermal relaxation of the annealed film changed significantly, and we needed three exponents with different relaxation times to fit the dependence $\Delta T_{e}\left(t\right)$ with sufficient accuracy. These time constants are also shown in Figure 3. The longest and middle ones are of the same order of magnitude as the times $\tau_{L}$ and $\tau_{S}$ measured in other samples. The shortest time constant of the annealed film $\tau_{0}$ is about 6–10 times smaller than $\tau_{S}$, and has a linear temperature dependence.

## 4. Discussion

Summarizing these observations, we conclude that the appearance of an additional relaxation time $\tau_{L}$ cannot be explained by surface effects. One may alternatively relate it to magnetic impurities in the bulk of the films. However, a previous experiment [45] has ruled out such a possibility for Cu films. It was found in Ref. [45] that Cu films with a very low concentration of magnetic impurities still exhibited long relaxation time. In contrast, a less pure silver (Ag) film had only shown a short relaxation time $\tau_{S}$, and its value was in good agreement with the predictions of free electron model. We show below that the origin of the multiscale thermal relaxation could be explained by the morphology of the films.

In order to analyze the two-scale relaxation of the electron temperature on a quantitative level, we propose a phenomenological model, in which the electrons in the Cu film are coupled to phonons and to an additional thermal reservoir, as shown in Figure 1. In this model, the time evolution of temperatures follows the equations

(2) $$\begin{array}{ccc} C_{e}\frac{dT_{e}}{dt} & = & -\Sigma V\left(T_{e}^{5}-T_{ph}^{5}\right)-\Sigma_{d}\left(T_{e}^{\alpha }-T_{d}^{\alpha }\right)+P_{H}\left(t\right),\\ C_{ph}\frac{dT_{ph}}{dt} & = & \Sigma V\left(T_{e}^{5}-T_{ph}^{5}\right)-\kappa A\left(T_{ph}^{4}-T_{b}^{4}\right),\\ C_{d}\frac{dT_{d}}{dt} & = & \Sigma_{d}\left(T_{e}^{\alpha }-T_{d}^{\alpha }\right). \end{array}$$

Here, $P_{H}\left(t\right)$ is the heating power, *A* is the contact area between the film and the dielectric substrate, κ is the constant characterizing thermal boundary conductance between the phonons in the film and in the substrate, $C_{ph}$ is the heat capacity of the phonons in the film, $C_{e}=\gamma VT_{e}$ is the heat capacity of electrons. $C_{d}$ is the heat capacity of the thermal reservoir, $T_{d}$ is its temperature, $\Sigma_{d}$ is the constant characterizing the coupling between electrons and the reservoir, and α is an unknown exponent, which we obtain from the fits. The value of this parameter may hint at the heat exchange mechanism between electrons and the additional thermal reservoir. For example, if the electronic system of the film is coupled to the additional thermal reservoir via electronic tunneling, then α=2. If phonons mediate the heat exchange, one expects α=4. In the latter case, the parameter $\Sigma_{d}$ depends on the acoustic properties of the interface between the copper film and the additional thermal reservoir, as well as on the contact area between them. The phonon heat capacity is usually very small, and one can put $C_{ph}=0$. Adopting this approximation and considering linearized versions of Equation (2), which are valid at sufficiently small $P_{H}$, we obtain $\Delta T_{e}\left(t\right)$ after an abrupt removal of the heating in the form of Equation (1) with the relaxation times and the pre-factor *a* having the form

(3) $$\begin{array}{ccc} \frac{1}{\tau_{L,S}} & = & \frac{1}{2}\left[\frac{1}{\tau_{d}}+\frac{1}{\tau_{t}}\right]\mp \sqrt{\frac{1}{4}{\left[\frac{1}{\tau_{t}}-\frac{1}{\tau_{d}}\right]}^{2}+\frac{C_{d}}{C_{e}+C_{d}}\frac{1}{\tau_{t}\tau_{d}}},\\ a & = & \frac{\tau_{d}^{-1}-\tau_{L}^{-1}}{\tau_{S}^{-1}-\tau_{L}^{-1}}\frac{\tau_{d}}{\tau_{S}}. \end{array}$$

The time $\tau_{d}$, appearing above, characterizes the relaxation between electrons and the additional thermal reservoir, $\tau_{d}^{-1}=\left(C_{d}^{-1}+C_{e}^{-1}\right)G_{d}$, and $\tau_{t}$ is the relaxation time of the electron temperature in the absence of the reservoir. It is given by the sum of two contributions:

(4) $$\begin{array}{ccc} & & \tau_{t}=\tau_{e-ph}+\tau_{ph-ph},\\ & & \tau_{e-ph}=C_{e}/G_{e-ph},\;\;\tau_{ph-ph}=C_{e}/G_{ph-ph}. \end{array}$$

In the above expressions, $G_{d}=\alpha \Sigma_{d}T^{\alpha -1}$ is the thermal conductance between electrons and the reservoir, $G_{e-ph}=5\Sigma VT^{4}$ is the thermal conductance between electrons and phonons, and $G_{ph-ph}=4\kappa AT^{4}$ is the thermal conductance between the phonons in the film and in the substrate. In Figure 2b we plot the times $\tau_{e-ph}$, $\tau_{ph-ph}$ and $\tau_{t}$, given by Equation (4), with the constants Σ = 2 ${\mathrm{nWK}}^{-5}$μ$m^{-3}$ and κ = 60 ${\mathrm{pWK}}^{-4}$μ$m^{-2}$ measured in an indepedent steady-state experiment [44]. We observe rather good agreement between the measured values of $\tau_{S}$ and the calculated times $\tau_{t}$.

Next, we invert Equations (3) and (4) and express the heat capacity of the thermal reservoir, $C_{d}$, and the thermal conductance, $G_{d}$, in terms of measured parameters,

(5) $$\begin{array}{ccc} C_{d} & = & \left[{\left(\tau_{L}-\tau_{S}\right)}^{2}/\tau_{L}\tau_{S}\right]a\left(1-a\right)\gamma VT, \end{array}$$

(6) $$\begin{array}{ccc} G_{d} & = & \left[C_{d}C_{e}/\left(C_{d}+C_{e}\right)\right]\left(\tau_{L}^{-1}+\tau_{S}^{-1}-\tau_{t}^{-1}\right). \end{array}$$

**Figure 4:**
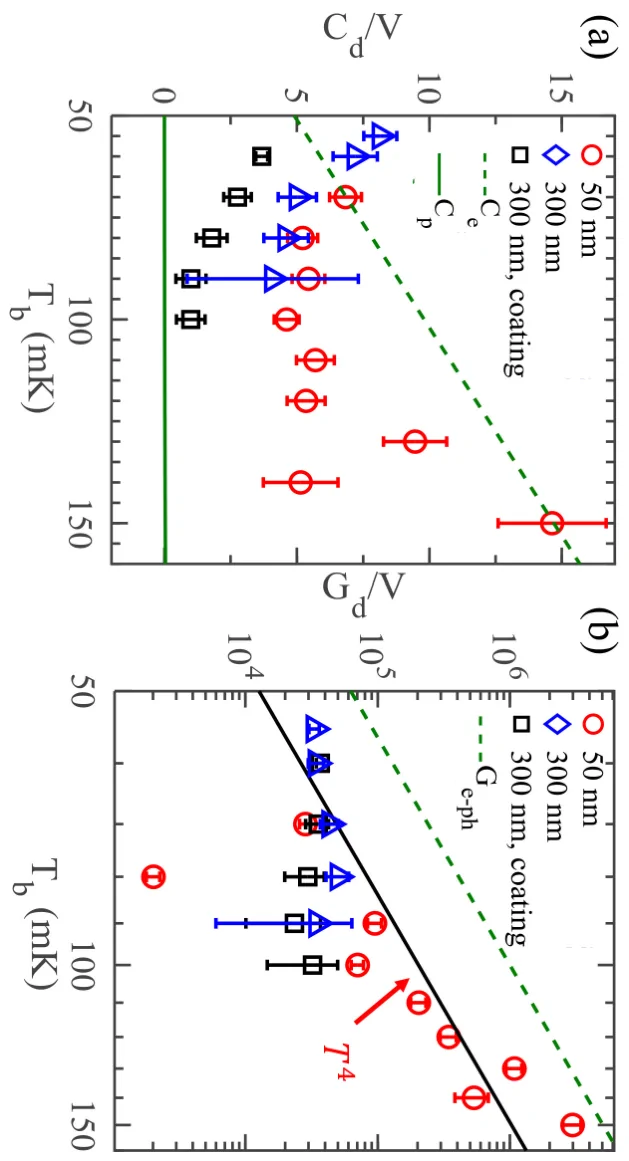
(**a**) Temperature dependence of the specific heat of the additional thermal reservoir coupled to electrons (5), i.e., heat capacity normalized by the volume of the absorber, $C_{d}/V$, for three different Cu films. Solid and dashed lines are the phonon and electron specific heats of the films, respectively. (**b**) Thermal conductance between the reservoir and the electrons (6) per unit volume, $G_{d}/V$. The dashed line shows the electron–phonon thermal conductance $G_{e-ph}/V$; the solid line is the fit of the data for the 50 nm thin film with $\propto T^{4}$ dependence.

These values are plotted for three different films in Figure 4a,b. The heat capacity $C_{d}$ does not exhibit clear temperature dependence in the explored temperature range. Its value is rather high and comparable to the heat capacity of electrons $C_{e}$. It is unlikely that low-concentration impurities would result in such a high value. One reason for that could be the existence of a significant number of grains in the film, which remain electrically decoupled from it. Such grains would form an additional thermal reservoir, which we have postulated in our model. In Figure 4b we show the temperature dependence of the thermal conductance $G_{d}$. For the two 300 nm films $G_{d}$ is almost constant, while for the 50 nm film it roughly follows power-law dependence with the exponent close to 4, which corresponds to α=5 in Equation (2).

Our hypothesis about electrically decoupled grains in the film as a reason for the long thermal relaxation time is supported by other experiments. Previously, experiments [45] have demonstrated that thermal relaxation in a uniform silver film follows a single exponential decay with the time constant expected from the theory, while two exponents are needed for a silver film with different grain orientations (See Supplementary Material). Experiments with copper films have shown that grains with different crystal lattice orientations relative to the film surface, which can be expressed in terms of Miller indices <ijk>, can simultaneously grow on ${\mathrm{SiO}}_{2}/\mathrm{Si}$ substrate [42,43]. At the beginning of the growth process a film with <111> orientation is formed. Subsequently, grains with <110> orientation are nucleated at the boundaries of the <111> grains, which results in the growth of <110> texture. Afterwards, <111> grains can again nucleate at the boundaries between <110> grains and so on. In the end, one obtains a sandwiched structure of alternating textures, which might be poorly coupled electrically, especially in our films, which were in contact with atmosphere between fabrication and measurement. It is also well known that electron–phonon scattering rates in bulk Cu are strongly anisotropic [37]. Since in thin films phonon wave vectors are parallel to the surface and since the Fermi velocity of electrons is much higher than the speed of sound, phonons predominantly interact with electrons moving perpendicular to the film surface. Thus, in grains with a <110> orientation, for example, the scattering rate should be close to that of electrons moving in the <110> direction in the bulk material. Bulk relaxation time in the <110> direction is, indeed, significantly longer than that in the <111> direction, as magnetic resonance experiments have shown [37]. In Figure 3b, we plot measured electron–phonon relaxation time in the bulk Cu in the <110> direction, $\tau_{\langle 110\rangle }=10^{-7}\times {\left(T/1K\right)}^{-3}$ s [37,38,39], and find that its value is indeed close to the measured $\tau_{L}$. Within this scenario, the high value of the pre-factor in front of the slowly decaying exponent observed for the 50 nm thin Cu film, a≈0.8, points to the dominant <110> texture. For the two 300 nm films, *a* drops from 0.5 at low *T* to 0 at higher *T* (See Supplementary Material), which hints at <111> as preferred orientation.

Though the above arguments qualitatively explain our findings, further experiments and more detailed theoretical modeling are required in order to fully understand heat relaxation mechanisms in thin Cu films. For example, thermal relaxation in the annealed 50 nm film can only be fitted with three exponents and, therefore, cannot be described by the simple model (2). On the other hand, the pronounced effect of annealing on thermal relaxation points at the importance of the grain structure of the film. Yet another unclear issue is the nature of the thermal coupling between conducting and electrically decoupled grains, which is described by the thermal conductance $G_{d}$. We expect such coupling to occur through tunnel barriers between the grains, which would result in $G_{d}\propto T$ temperature dependence, but the observed $G_{d}\propto T^{4}$ scaling differs from that. We have also considered an alternative thermal model, in which the additional reservoir couples to film phonons instead of electrons. However, this model results in much smaller values of the pre-factor *a* than the observed ones.

## 5. Conclusions

In summary, we have investigated dynamic thermal relaxation in Cu and Ag films at sub-kelvin temperatures. In contrast to uniform Ag thin films, which are well described by the free electron model [45,46], thermal relaxation in granular Cu and Ag films is complicated and characterized by two or more relaxation times. One possible explanation for this effect could be the existence of a thermal reservoir consisting of the grains weakly electrically coupled to the current-carrying electrons. Our experiment refines the understanding of non-equilibrium thermal transport in mesoscopic metallic structures and should help to further optimize the performance of the devices utilizing thermal effects and operating at low temperatures.

## Data Availability

The original contributions presented in this study are included in the article/supplementary material. Further inquiries can be directed to the corresponding author.

## Supplementary Materials

The following supporting information can be downloaded at: https://www.mdpi.com/article/10.3390/e28080908/s1.
