# Supplementary material for: Dynamic Thermal Relaxation in Metallic Films
Source: Entropy (Basel). 2026 Aug 13;28(8):908. doi: 10.3390/e28080908 (PMC13511967; doi:10.3390/e28080908)
Supplement: Supplementary file 1 [file entropy-28-00908-s001.zip › entropy-4439353-supplementary.pdf]

Supplemental Material for "Dynamic Thermal Relaxation in Metallic Films"

Libin Wang,[1,*] Dmitry Golubev,[2] Yuri. M. Galperin,[3] and Jukka. P. Pekola[1]
[1]*QTF Centre of Excellence, Department of Applied Physics, Aalto University, FI-00076 Aalto, Finland*
[2]*HQS Quantum Simulations GmbH, Rintheimer Str. 23, 76131 Karlsruhe, Germany*
[3]*Department of Physics, University of Oslo, PO Box 1048, Blindern, 0316 Oslo, Norway*

CHARACTERIZATION OF JOSEPHSON JUNCTION THERMOMETER

The switching from the superconducting to the resistive state of a proximity Josephson junction (PJJ) occurs stochastically due to both thermal and quantum fluctuations. In order to define the switching current I_{sw} we have calibrated the thermometer. We have probed the switch by sending a pulse train with N pulses with the fixed probing current I_p, and recorded the number of switchings, n, by monitoring the voltage across the PJJ. The switching probability at a fixed bias current is defined as $P = n/N$. We define the stwitching current, I_{sw}, as the bias current at which $P = 50\%$. Temperature dependence of I_{sw} is shown in Figure. S1 (a). No saturation of I_{sw} is observed down to 60 mK.

To characterize the performance of the PJJ thermometer we define the temperature resolution of the thermometer,

$$\epsilon_T = \frac{\Delta I_p}{dI_{sw}/dT}, \tag{S1}$$

where $\Delta I_p = I_p(P = 90\%) - I_p(P = 10\%)$ is the width of the switching distribution. For our underdamped PJJ, ΔI_p is around 20 nA. The calculated temperature resolution of the thermometer is shown in Figure. S1 (b). In the temperature range explored, ϵ_T is around 0.1 mK for a probing pulse with the width of 2 μs. We have used heating signals, $I_H(t)$, consisting of two pulses (pulse width 500 μs) of opposite polarities to heat film electrons. Figure. S1 (c) shows the measured switching current changes $\Delta I_{sw}^{+(-)} = I_{sw}^{+(-)} - I_{sw}^{+(-)}(t = 0)$ for both polarities. Here, +(-) indicates the direction of the probing current. Overlapping of $\Delta I_{sw}^{+(-)}$ confirms the accuracy of the measurements. In addition, we have calculated the switching current offset $I_{sw}^{+} - I_{sw}^{-}$ and plotted it in Figure. S1 (c) with the blue circles. The offset fluctuates around an averaged value and dos not depend on whether I_H is turned on or off, thus indicating that the heating current flowing to the temperature probing line is negligible.

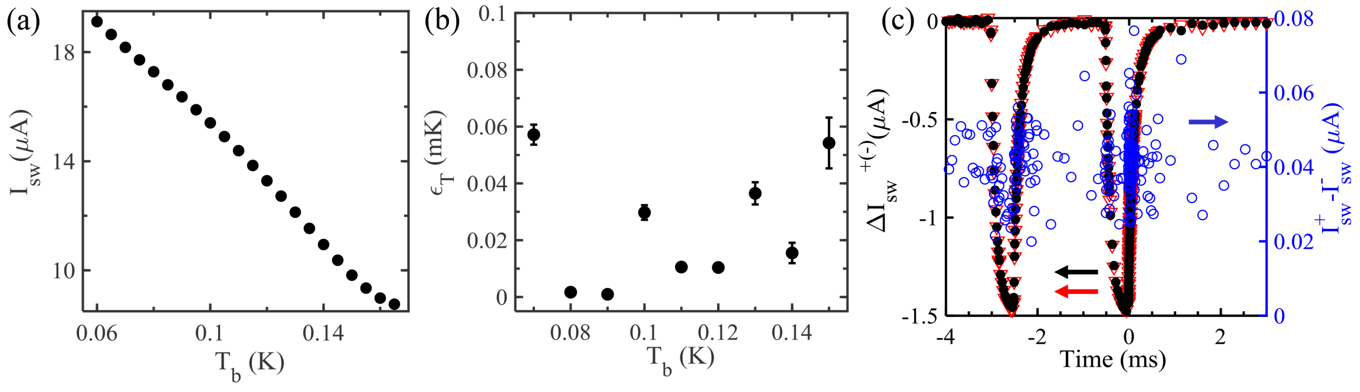

Figure S1: Characterization of the PJJ thermometer. (a) I_{sw} as a function of bath temperature T_b, which is used as the temperature calibration of the PJJ thermometer. (b) Temperature resolution ϵ_T of the PJJ thermometer in the temperature range explored. (d) Switching current changes $\Delta I_{sw}^{+(-)}$ and the offset as a function of the time interval between I_H and I_p.

EFFECT OF HEATING PULSE AMPLITUDE ON THERMAL RELAXATION

In the experiment we have varied the magnitude of the heating pulses. We show the measured electron temperature with I_H varying from 0.1 to 0.2 μA at $T_b = 70$ mK in the inset of Figure. S2 (a). The thickness of the Cu film in this measurement was 300 nm and its top surface has been coated with 5 nm of Al. The steady-state temperature

increment $\Delta T_{es} = T_{es} - T_b$ grows from 6 to 18 mK with I_H increasing from 0.1 to 0.2 μA. The time dependence of the normalized temperature change $\Delta T_e(t)/\Delta T_{es}$ is shown in Figure. S2 (a). Fitting the relaxation process with Eq. (1) in the main text, we derive the two thermal relaxation times τ_S and τ_L and plot them as a function of temperature in Figure. S2 (b). To derive Eq. (S1) in the main text we have assumed the temperature increase ΔT_e is much smaller than T_e. If high heating power is applied, this assumation is no longer valid, and, for example, the time τ_S extracted from the fits becomes smaller because it decreases at higher temperatures (see Figure. S2 (b)). However, the long relaxation time τ_L is almost not affected by the heating magnitude. For the data shown in the main text the maximum temperature rise was about $\Delta T_{es} = 19$ mK and it quickly dropped below 5 mK at higher bath temperature. Thus, we can neglect the influenece of heating amplitude on the relaxation times.

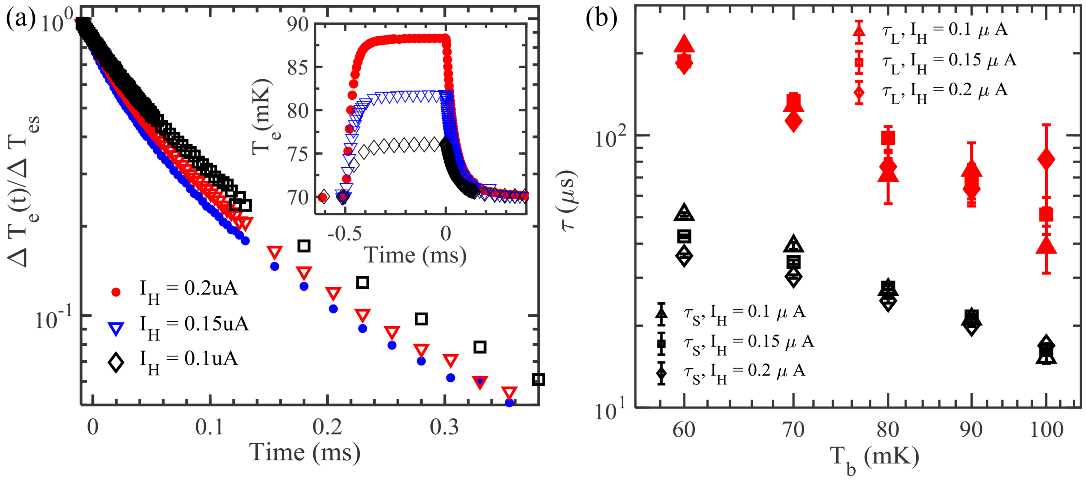

Figure S2: Impact of heat magnitude on thermal relaxation. (a) Normalized electron temperature increases $\Delta T_e(t)/\Delta T_{es}$ as a function of time interval between probing current pulse I_p and heating curent pulse I_H. The heating current pulse I_H varies from 0.1 (bottom, diamonds) to 0.2 μA (top, triangles). Inset: Electron temperature in response to I_H with varying magnitude, labels are the same as in the main plots. (b) Temperature dependence of the derived two thermal relaxation times τ_L (solids) and τ_S (hollows).

EFFECT OF ANNEALING ON THERMAL RELAXATION

We have measured a 50 nm thick annealed Cu film in order to study the effect of grain size and grain interface on the dynamic thermal relaxation. The annealing of the film was carried out in N_2 ambient at 350°C for 20 mins. Clean Al contacts to the Cu film have been fabricated after the annealing. SEM images of the Cu film before and after annealing are shown in Figure. S3. Growth of the grain sizes after annealing is evident.

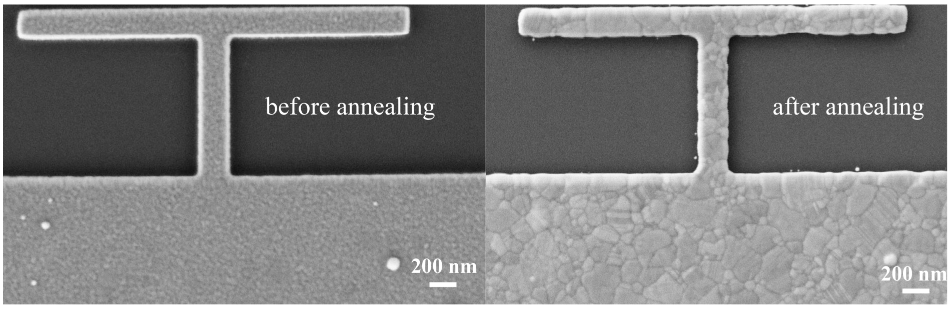

Figure S3: SEM images show the grain growth after film annealing.

Interestingly, we have also found that thermal relaxation process is significantly affected by an nealing. Fi gure S4 (a) shows the experimental results for the annealed 50 nm Cu film. We find that the thermal relaxation process cannot be well fitted with the double exponential decays defined in Eq. (S1) of the main text. Instead, one needs three

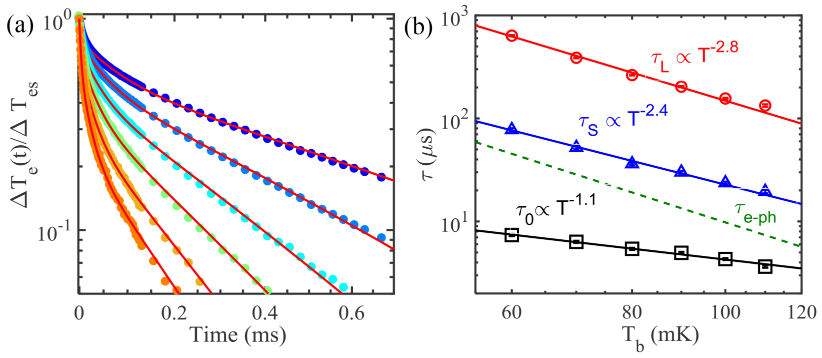

Figure S4: Measurement results of the annealed 50 nm Cu film. (a) $\Delta T_e/\Delta T_{es}$ as a function of time. T_b varies from 60 mK (up-blue) to 120 mK (bottom-red) with 10 mK temperature interval. Red lines are fits with Eq. S2. (b) Temperature dependence of the three thermal relaxation times. Solid lines are the fits to their temperature dependence. Dashed line is the calculated e-ph thermal relaxation time with equation $\tau_{e-ph} = \gamma/5\Sigma T^{-3}$, Sommerfeld constant $\gamma_{Cu} = 98$ $\mathrm{JK^{-2}m^{-3}}$, electron-phonon thermal coupling constant $\Sigma_{Cu} = 2$ $\mathrm{nWK^{-5}m^{-3}}$.

exponents in order to fit the data. In Figure. S4, we plot the $\Delta T_e(t)/\Delta T_{es}$ for the annealed film together with fits with the expression

$$\Delta T_e/\Delta T_{es} = ae^{-t/\tau_L} + be^{-t/\tau_S} + (1-a-b)e^{-t/\tau_0}, \tag{S2}$$

where a, b are constants. The extracted three time constants τ_L, τ_S and τ_0 are plotted in Figure. S4 (b). We find that the longest two time constants (τ_L, τ_S) are on the same order of the two time constants in the films not subject to annealing. The shortest time τ_0 is about 6 - 10 times smaller than τ_S, as discussed in the main text. Temperature dependence of the three times is shown in Figure. S4 (b). Measurement results for the annealed film cannot be explained by the thermal model introduced in the main text. However, the experimental results clearly indicate the importance of the grain structure of the film for thermal relaxation. One can speculate, for example, that the annealed film contains grains with three different orientations, which may explain the origin of three relaxation times.

PRE-FACTOR a EXTRACTED FROM THE FITS

In the main text, we have introduced the thermal model considering film electrons thermally coupled to the phonons and to an additional thermal reservoir. Within this model, the dynamic relaxation process of Cu films is described by Eq. (S1) in the main text. Fitting the experimetal data with Eq. (S1), we obtain the two time constants τ_L and τ_s, as well as the prefactor a in front of the slowly decaying exponent. In Figure. S5, we show its temperature dependence obtained for the two 300 nm Cu films and the as-deposited 50 nm Cu film. For the annealed 50 nm Cu film, one needs to used three exponents to fit the data, as shown in the previous section. We find that for the 50 nm Cu film a remains close to 0.8 without obvious temperature dependence. However, for the two 300 nm Cu films a drops from 0.5 at 55 mK to close to 0 at 100 mK. Origin of such temperature dependence remains unclear and requires further investigation. The high value of a observed in 50 nm Cu film suggests that the dominant texture is this film was $\langle 110 \rangle$. In contrast, in the two 300 nm Cu films with $a < 0.5$, the $\langle 111 \rangle$ texture probably dominates.

THERMAL RELAXATION IN A SILVER FILM

Previous experiments [1] have shown that in the uniform Ag film thermal relaxation exhibited a single relaxation time, which has been in agreement with the prediction of the free electron model. We have carried out an additional experiment with a 50 nm silver film having granular structure. Figure S6 (a) shows the transmission electron microscopy (TEM) image of that film. In constrast to the experiment [1], the film image clearly shows grains of different orientations. We have found that the electron temperature relaxation in the granular Ag film is a double exponential decay similarly to copper films. It is illustrated in Figure. S6 (b). In the same way as for the Cu films discussed in the main text, the value of the shorter relaxation time τ_S is close to the calculated e-ph thermal relaxation time τ_{e-ph}, while the longer relaxation time τ_L is about one order of magnitude larger. We believe that the difference between the

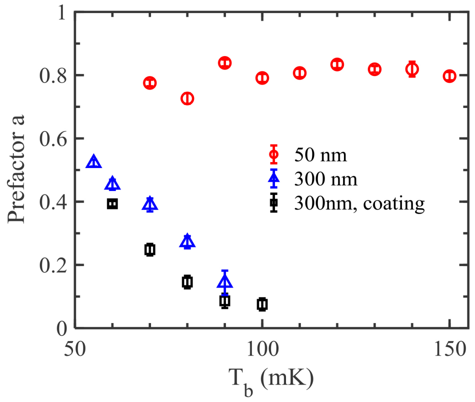

Figure S5: Temperature dependence of the prefactor a derived from the fits to experiment data with Eq. (S1) in the main text.

two times can also be explained in the same way. Indeed, magnetic resonance experiments [3, 4] have demonstrated strong anisotropy of the e-ph scattering in silver. In the inset of Figure. S6 (b) we show the e-ph relaxation time measured in thick silver films with <110> orientation averaged over magnetic field direction, $\tau_{\langle 110 \rangle} \approx 10^7 (T/1\mathrm{K})^3$ Hz (note that this value coincides with the one measured in copper), and find that it is close to the measured τ_L. Thus, our measurement of the silver film further emphasize the importance of the grain texture of the film for thermal relaxation.

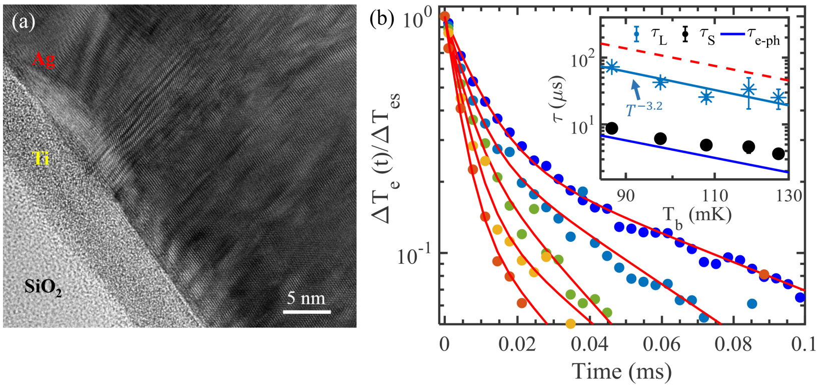

Figure S6: (a) TEM image shows the Ag film with grains of different orientations. The Ag film is evaporated on a SiO_2/Si substrate with 5 nm of Ti in-between. (b) Normalized electron temperature $\Delta T_e/\Delta T_{es}$ as a function of time. $T_b = 87$, 97, 108, 119, 127 mK from right to left. Red lines are fits with Eq. (S1) in the main text. Inset: temperature dependence of τ_S (dots) and τ_L (stars) derived from the fits. $\tau_{e-ph} = \gamma/5\Sigma T^3$ is the calculated e-ph thermal relaxation time. For Ag, we used $\Sigma_{Ag} = 3$ nWK$^{-5}\mu$m^{-3}[1] and $\gamma_{Ag} = 63$ JK^{-2}m^{-3} [2]. Red-dashed line show the e-ph relaxation time of electrons moving in <110> direction, $\tau_{<110>} = 1.7\times10^{-7}\,(\mathrm{T}/1\mathrm{K})^{-3}$ s [3].

THERMAL EQULIBRIUM TIME BETWEEN THE HEATER, THE THERMOMETER AND THE PAD

The thermal equilibration time between the heater, the thermometer and the pad is estimated as $\tau_{eq}^{ij} = C_i/G_{ij}$, where C_i is the heat capacity of the heater and G_{ij} is the heat conductance of the bridge between the heater, the therometer and the large pad. For metallic films at low temperatures, C_i is dominated by electronic heat capacity and is estimated from the free electron model as $C_i = \gamma V T$, where V is the volume. Heat conductance G_{ij} is dominated by the electrons and one can estimate it using the Wiedemann-Franz law as $G_{ij} = LTG_{el}$, where G_{el} is the electronic conductance and $L = 2.44 \times 10^{-8}$ WΩK^{-2} is the Lorenz number. The resistivity of the narrow Cu wire is measured from the heater resistance of about $\rho = 1\times10^{-8}$ Ωm. For a typical bridge between the heater, the thermometer and the pad with dimensions of 700 nm $\times$ 250 nm $\times$ 50 nm (length $\times$ width $\times$ thickness), $G_{el} \approx 2$ S. At temperature of 0.1 K, the estimated $\tau_{eq}^{ij} \approx 1$ ns, which is much shorter than the measured time constants. Thus, the equilibration

between the heater and the thermometer cannot explain the appearance of additional time constants.

REFERENCES

[1] Viisanen, K.L.; Pekola, J.P. Anomalous electronic heat capacity of copper nanowires at sub-Kelvin temperatures. *Phys. Rev. B* **2018**, *97*, 115422. https://doi.org/10.1103/PhysRevB.97.115422.

[2] Kittel, C. *Introduction to Solid State Physics*, 8th ed.; Table 2; Wiley: Hoboken, NJ, USA, 2005. https://www.wiley.com/en-us/shop/general-physics/introduction-to-solid-state-physics-8th-edition-p-9780471415268

[3] Gasparov, V.A. Anisotropy of the probability of electron-phonon scattering in silver. *J. Exp. Theor. Phys.* **1975**, *68*, 2259–2270. https://jetp.ras.ru/cgi-bin/dn/e_041_06_1129.

[4] Johnson, P.B.; Goodrich, R.G. Anisotropy of the electron-phonon collision frequency on the Fermisurface of silver. *Phys. Rev. B* **1976**, *14*, 3286–3295. https://doi.org/10.1103/PhysRevB.14.3286.
